# An Opportunistic Pathogen Afforded Ample Opportunities: Middle East Respiratory Syndrome Coronavirus

**DOI:** 10.3390/v9120369

**Published:** 2017-12-02

**Authors:** Ian M. Mackay, Katherine E. Arden

**Affiliations:** 1 Public and Environmental Health Virology, Forensic and Scientific Services, Department of Health, Queensland Government, Coopers Plains, 4108 QLD, Australia; 2 Child Health Research Centre, The University of Queensland, South Brisbane, 4101 QLD, Australia; k.arden@uq.edu.au

**Keywords:** Middle East respiratory syndrome, MERS, coronavirus, comorbidity, infection, camel, zoonosis, emerging virus

## Abstract

The human coronaviruses (CoV) include HCoV-229E, HCoV-OC43, HCoV-NL63, and HCoV-HKU1, some of which have been known for decades. The severe acute respiratory syndrome (SARS) CoV briefly emerged into the human population but was controlled. In 2012, another novel severely human pathogenic CoV—the Middle East Respiratory Syndrome (MERS)-CoV—was identified in the Kingdom of Saudi Arabia; 80% of over 2000 human cases have been recorded over five years. Targeted research remains key to developing control strategies for MERS-CoV, a cause of mild illness in its camel reservoir. A new therapeutic toolbox being developed in response to MERS is also teaching us more about how CoVs cause disease. Travel-related cases continue to challenge the world’s surveillance and response capabilities, and more data are needed to understand unexplained primary transmission. Signs of genetic change have been recorded, but it remains unclear whether there is any impact on clinical disease. How camels came to carry the virus remains academic to the control of MERS. To date, human-to-human transmission has been inefficient, but virus surveillance, characterisation, and reporting are key to responding to any future change. MERS-CoV is not currently a pandemic threat; it is spread mainly with the aid of human habit and error.

## 1. Introduction

Acute respiratory tract infections (ARTIs) are a frequent cause of morbidity and mortality and a common reason for both outpatient visits and hospitalisations. Among humans worldwide, RNA viruses the agents that cause ARTI most frequently—usually a self-limiting upper respiratory tract syndrome. Coronaviruses (CoVs) are recombining, enveloped viruses with long positive-sense RNA genomes. They are ancestrally zoonotic in origin, having adapted to bind a diverse range of cellular receptors [1]. Four human coronaviruses (HCoVs) have evolved, initially from bats, camelids, and rodents, to become distinct global, endemic, seasonal pathogens causing mild to moderate ARTI among humans [2,3].

The HCoVs occupy two of four genera in the subfamily *Coronavirinae* [4]. *Human coronavirus 229E* and *Betacoronavirus-1* subspecies *HCoV-OC43* have been known for more than 50 years, while *Human coronavirus NL63* and *Human coronavirus* HKU1 were first characterised in 2004 and 2005, respectively.

A *Severe acute respiratory syndrome-related coronavirus* (SARS-CoV) briefly emerged into the human population during 2002–2004 but was controlled and is not known to circulate today. Its brief but severe emergence sparked renewed study of CoVs. In 2012, another novel, severely pathogenic CoV was identified in the Kingdom of Saudi Arabia (KSA); 80% of over 2000 human cases have been recorded over five years [5]. Both SARS-CoV and the new Middle East respiratory syndrome coronavirus (MERS-CoV; belonging to the species *Middle East respiratory syndrome-related coronavirus*) evolved from ancestral, but different, bat CoVs. Travel history and laboratory analysis would be required to differentiate MERS from SARS, if it still occurred [6]. To date, most MERS cases have been limited to countries in the Arabian Peninsula with rare travel-related spillovers. One case precipitated a large healthcare multi-facility outbreak in the Republic of Korea in 2015.

A confirmed case of MERS occurs when a person, irrespective of signs or symptoms, has a laboratory-reported MERS-CoV infection. A probable case requires a minimum of a clinically diagnosed acute febrile disease, an epidemiologic link, and a partial laboratory diagnostic profile [7].

We briefly review recent literature and the current understanding of MERS and MERS-CoV highlighting some knowledge gaps.

## 2. Virus Attachment

**Figure 1:**
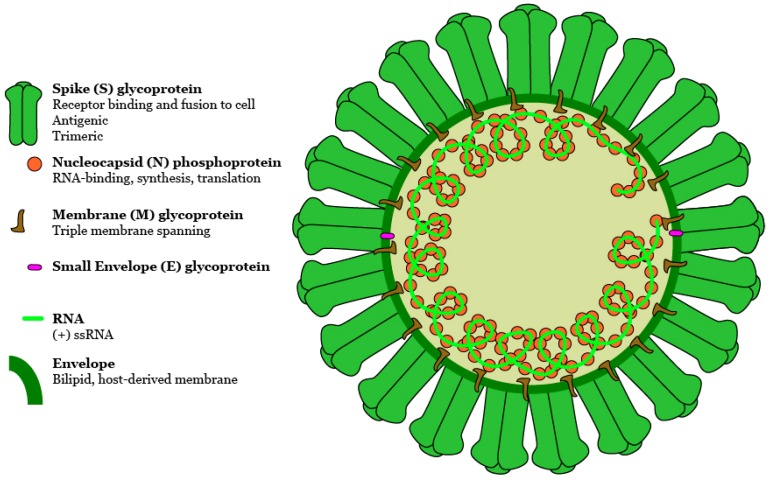
Schematic of a predicted MERS-CoV virion. Along with membrane and envelope transmembrane proteins, the spike glycoprotein protrudes from a host cell-derived lipid bilayer, giving the virion a distinctive appearance. Positive-sense viral RNA is associated with nucleocapsid phosphoprotein in a helical structure. DOI:10.6084/m9.figshare.5639320.

The viral receptor for MERS-CoV is a transmembrane glycoprotein called dipeptidyl peptidase-4 (DPP-4), which has a wide tissue distribution in humans, including on alveolar cells of the lower respiratory tract and in the small intestine [8,9]. DPP-4 interacts with the MERS-CoV at its receptor-binding domain (RBD) within the spike fusion protein (Figure 1). DPP-4 expression is upregulated in the lower respiratory tract of those with poor lung function, in the lower respiratory tract of those with untreated asthma, and in soluble form in the plasma of obese patients [8,10,11,12]. Whether blood borne DPP-4 interferes with MERS-CoV or, through reversible binding, contributes to its systemic distribution of MERS-CoV during serious disease, are questions worthy of further investigation. Studies of the upper respiratory tract have found little to no sign of DPP-4 expression levels or tissue distribution, although one study identified DPP-4 enzymatic activity, suggesting it is present [10,13,14]. It may be useful to expand the search for DPP-4 expression to tissues indicated by cases with different disease states. Co-infections, especially with viruses known to inflame the lower respiratory tract and trigger asthma exacerbations such as rhinoviruses are also of interest. MERS-CoV may also use sialic acids as a low-affinity but selective cellular receptor to aid attachment and entry into DPP-4 positive cells [15].

## 3. Disease and Immunity

MERS is most well characterised as a respiratory disease of humans. Extensive inflammation and immune evasion are hallmarks of severe disease ascribed to CoVs [16,17]. Among confirmed MERS cases, fever, cough, and dyspnoea are the most common clinical manifestations [18,19]. The mean incubation period for MERS is between 2 and 13 days. Longer periods are associated with a reduced risk of death [20,21,22]. MERS-CoV infection also results in mild and subclinical outcomes.

The typical MERS case is a Saudi male aged between 21 and 60 years, often presenting to a hospital with pneumonia, or worse [18,23,24]. Severe MERS occurs most frequently among those with comorbidities including diabetes mellitus, cirrhosis, and others affecting respiratory, renal, and cardiac systems [23,25,26]. Downregulation of innate immune response mediators associated with some of these disorders may be related to the severity of MERS [27]. It may be that the high frequency of severe MERS reflects elevated prevalence of certain comorbidities in the Middle East region [27]. Comorbidities did not feature among SARS cases, as they have among cases of MERS; MERS-CoV is a highly opportunistic pathogen.

In June 2015, an outbreak in the Republic of Korea found confirmed cases presented with fever, cough, and upper respiratory tract signs and symptoms, progressing within a week to lower respiratory tract distress with lymphopenia and elevated liver enzymes [23,24,27,28]. MERS can progress to acute respiratory distress syndrome and multiorgan system failure requiring intensive supportive care [23,24,29]. Extra pulmonary infection does occur, likely resulting from haematogenous transport of virus, which is an area in need of further study [9,30]. Similarly, little is known about sequelae following MERS-CoV infection; in one study, those who survived acute respiratory distress syndrome were well one year later [19]. Another study identified delayed neurological manifestations during treatment of MERS cases, but lacked long-term follow-up [31].

Death occurs among 30–40% of MERS cases; approximately 40% of cases in the KSA, and 20% of cases in the Republic of Korea, where mortality ranged from 7% among younger age groups to 40% among those aged 60 years and above. Among studies of fatal cases, death occurred between 5–15 days after symptom onset [18,21,32,33]. Because the extent of subclinical or mild infections in the community remains unquantified, mortality figures may be overestimates [34].

MERS-CoV variants exist as a single antigenic group in lineage C of the genus *Betacoronavirus* [35,36]. When MERS-CoV infection is subclinical or less severe, humoral immune responses may be weak, delayed, short-lived, or undetectable [37,38,39,40,41]. Humans can still be reinfected if they have pre-existing neutralising antibodies, similarly to what is seen in camels [41]. However, survivors of symptomatic MERS do develop antibodies, including neutralising antibodies, which decline but persist for 1–3 years [37,42]. Whether these antibodies prevent future infection remains to be examined. Those with mild or subclinical MERS still develop CD8^+^ T-cell responses, and survivors of more severe MERS, including those who do not mount an antibody response, develop both CD8^+^ and CD4^+^ T-cell responses [43].

## 4. Detection Methods and Gaps

Robust laboratory-based real-time RT-PCR diagnostic tools were described immediately after MERS-CoV was discovered and remain reliable [44]. Several different research-based antibody detection protocols have also been reported for human and animal studies [45]. Molecular and serological kits are commercially available [46]. While current molecular methods are rapid, there are many steps that combine to delay publication of a final test result; from the initial decision to request a MERS-CoV test, to sample collection, nucleic acid extraction, PCR, additional sampling, repeat testing, and reporting processes. Molecular, rapid, and sensitive point-of-care tests (POCTs) are not available but would help support infection control in healthcare settings.

The World Health Organisation recommends testing of appropriate samples for MERS-CoV RNA using real-time RT-PCR methods with subgenomic sequencing to confirm screening results, as necessary [47]. Repeat testing is often required [17,48]. Virus isolation by culture is not a recommended tool. Detecting antibodies against MERS-CoV may be useful to identify a seroconversion that can define a probable case when confirmation by RT-PCR has been unsuccessful or impossible [47].

Mild and subclinical MERS cases are reported among younger people including healthcare workers and children [17,49,50]. It remains unclear what proportion of MERS-CoV infections are truly subclinical after one study found many were initially incorrectly classified [51]. Difficulty identifying a useful diagnostic antibody response in mild and subclinical disease means seroprevalence studies likely under-report the history of MERS-CoV [40]; MERS-CoV specific CD8^+^ testing may be helpful [43]. The usefulness of serology needs clarification.

## 5. Virus Origins: Camel to Human with a Bat Ancestry

Humans are incidental hosts of MERS-CoV. The principal natural reservoir host of MERS-CoV is the dromedary camel [16]. Among camels, MERS-CoV infection manifests in a manner similar to that in which the common cold manifests in humans. The camel upper respiratory tract expresses high levels of the DPP-4 receptor [13]. The study of MERS-CoV highlights the need for collaboration between politics and diverse basic and applied fields of research at the human–animal interface—a One Health approach. Camel trade plays a central role in the movement of infected hosts around the Arabian Peninsula. Pakistan and North, West, and East Africa all harbour MERS-CoV-infected camels [52,53,54,55]. Alpacas, llamas, and pigs are potential hosts according to experimental evidence [56,57,58]. In addition, alpacas have been found to be naturally infected [59].

The virus genetically detected or biologically cultured from humans is nearly identical to that isolated from camels and, in some instances, has been used to identify transmission routes. Signs of virus change have appeared with continued surveillance over time. Recombination has been identified, and variation in the region of the tropism-defining spike protein in a divergent Egyptian camel MERS-CoV variant [60] has been associated with reduced infectivity in vitro, compared to human and less divergent camel MERS-CoV variants [61]. Nearly 300 human and animal MERS-CoV genome sequences have been produced from MERS-CoV infections since 2012, but few studies of the functional impact of identified mutations have been conducted. Among MERS-related coronaviruses [62], there may now exist three conspecific viruses: the camel MERS-CoV and the bat CoVs, BtVs-BetaCoV/SC2013 [63] and PREDICT/PDF-2180 [64], each with related but distinct genomes and divergent spike genes. Ongoing viral surveillance and characterisation is essential to ensure variants with increased efficiency in attachment, fusion, or replication do not emerge unnoticed. Surveillance of human respiratory illness in MERS-CoV-positive camel countries outside of the Arabian Peninsula is currently lacking [52].

## 6. Transmission: Known Unknowns

According to the KSA Ministry of Health [65], nearly half of all MERS cases are classified as primary cases: zoonoses originating from direct or indirect contact with infected dromedary camels, or from an unidentified source which had no link to any other (known) human case. The precise mechanism by which MERS-CoV spreads from camels to humans is unknown but is not essential for enacting precautions to reduce exposure to infected animals. Secondary cases make up slightly more than half of all MERS cases, mostly resulting from exposure associated with a healthcare facility.

Infectious MERS-CoV is presumed to be found in droplets [59] but modelling has also suggested the possibility of airborne spread [45]. Virus remains viable for at least 48 h on plastic and steel surfaces, presumably underpinning the extensive contamination of air and surfaces in hospitals housing patients with MERS [66,67,68,69,70,71]. The virus appears sensitive to standard heat and chemical inactivation measures [72,73].

Antibodies to MERS-CoV have been found in camel sera as far back as 1983. In each animal, antibodies to MERS-CoV are short-lived and do not prevent reinfection [52,74,75].

Human contact with camels is often associated with the collection, preparation, and ingestion of camel milk or meat. Female camels, especially those bred for milking, have the highest rates of MERS-CoV seropositivity; MERS-CoV RNA has been detected in milk from one study and virus was found to be stable after being spiked into milk samples from another study [53,76,77]. Camels in larger herds have higher rates of seropositivity compared with smaller herds [52,53]. Female and young camels also have higher rates of MERS-CoV RNA than older and male camels [53]. While no evidence for human infection resulting from ingestion has been presented, it has been found that experimentally inoculated human intestinal cells and organoids can host productive MERS-CoV infection and that MERS-CoV can remain infectious after transit through gastric acids [9]. Further, intestinal, respiratory, and neurological infection follows intragastric inoculation of DPP-4 transgenic mice [9]. Whether camel milk and meat actually contain a suitable infectious dose to cause intestinal infection of humans is yet to be determined [78]. It seems likely that the processes of milking and butchery may contaminate surfaces and generate infectious droplets that include sufficient inoculum from which a human infection could result via inhalation or self-inoculation. It is not known whether the eyes act as a portal for MERS-CoV entry.

Since the majority of human-to-human MERS-CoV infections are associated with healthcare, improved infection control and prevention is considered key for preventing outbreaks among humans not at occupational risk of exposure to infected animals [79]. In the outbreak in the Republic of Korea, 5 of 186 cases were responsible for 83% of transmission events; most new cases did not result in any identified onward transmission; the reproduction (R_0_) number was calculated as 3.9 and 1.9–6.9 from selected KSA outbreaks [80]. Three of these five cases were coughing; prolonged exposures, crowding, and large numbers of contacts were important factors for disproportionate virus transmission [81,82,83].

The role, if any, for mild or subclinical MERS-CoV infections in maintaining the virus in the human population has not been convincingly addressed. A healthcare worker found to shed viral RNA for more than five weeks in the absence of disease adds urgency to the need for such studies [84]. None among 82 contacts of a mildly symptomatic MERS-CoV-infected healthcare worker seroconverted, but there was no mention of whether the index case seroconverted [85]. In a KSA hospital outbreak investigation, contacts of subclinical MERS-CoV-infected healthcare workers became RT-PCR positive, suggesting transmission was a possibility [17]. Some studies report very rare camel contact among human cases and no history of contact with other MERS-CoV-infected humans, and this raises the question of how these primary cases acquire infection [18]. Community spread and subclinical transmission need more attention.

The Hajj pilgrimage, an annual mass gathering in the KSA, provides many opportunities for MERS-CoV to transmit and then spread globally. However, it is rhinoviruses, influenza viruses, and other seasonal respiratory viruses that have, to date, driven the bulk of respiratory disease associated with the Hajj. This indirectly reinforces that MERS-CoV does not transmit efficiently among humans outdoors [86]. In hospital environments, healthcare workers and other patients and carers who experience prolonged exposure to infectious cases, in the absence of suitable personal protective equipment (PPE), are those who usually become infected. There have been examples of the 20/80 rule, whereby relatively few infected individuals are responsible for a disproportionate number of new cases [83]. Insufficient cleaning of room surfaces, inadequate room ventilation, and overcrowding have also been suggested to drive indoor MERS-CoV transmission.

## 7. Prevention and Treatment

No specific antiviral or licensed vaccine is available for a CoV that infects humans, but a range of candidates exist. Even if MERS-CoV infection is rare, transmits poorly, and does not evolve to become a pandemic threat, it serves in a useful role to drive vaccine research of other CoVs, both current and yet to emerge. For cases in healthcare facilities, improving hand hygiene, the use of PPE (gloves, gown, respiratory, and eye protection [87]), and surface cleaning can help disrupt transmission, as can rapid triage of febrile patients with respiratory signs and symptoms. To prevent MERS-CoV infection from dromedary camels, precautions include avoiding contact with camel nasal secretions, cooking camel meat, and pasteurising camel milk until further studies better quantify the risk attached to each of these potential pathways.

Vaccines to prevent CoV disease require both humoral and cellular immunity [88]. Because airway immune responses may be key to preventing the establishment of human MERS-CoV infection, localised deposition of an aerosolised vaccine could prove useful [88]. A number of vaccine platforms and payloads have proceeded although progress has been challenged by the need for animal models that suitably reconstitute human lower respiratory tract disease to show evidence of any preventative effect [89,90]. Some candidates have progressed to clinical trials [91,92]. The spike protein and RBD elicit neutralising antibody responses and have been employed as the payload for a number of platforms [88,93,94] including DNA vaccines [95], modified vaccinia virus Ankara [96,97], measles virus [97,98,99,100], and human- [26,101] and chimpanzee-adenovirus-based vectors [99]. There are also Venezuelan equine encephalitis replicons expressing nucleocapsid [101], nanoparticles [102], and structural and non-structural deletion mutants of MERS-CoV [103].

Vaccination of camels is likely to be the most rapid, least expensive, and best intervention for preventing rare spillovers that then become amplified by humans in healthcare settings. Successes have been reported, but the approach is challenged by the problem that camels are naturally reinfectable with MERS-CoV, even in the presence of a high titre of neutralising antibodies [52,74,104]. To date, camel vaccines reduce viral load but do not prevent virus shedding [104,105]. Human vaccines could target the occupational at-risk groups, which include healthcare, farm, barn, market, and slaughterhouse workers [56,88,106]. More widespread application of a MERS vaccine at this time does not seem warranted.

The rarity of seropositive donors, sometimes low antibody titres, and a lack of clinical evidence have made the use of convalescent sera from recovering MERS patients a possibility for treatment, but one with significant limitations [39]. Instead, human monoclonal antibodies targeting the RBD and polyclonal antibodies may provide treatment options for those at risk of severe outcomes [107,108,109,110]. Clinical trials are awaited.

Early control of viral replication is important and administration of interferon (IFN) β1b or a ribavirin and IFN α2b combination within hours initially showed promise. Their practical use in humans is challenging because, if not infected while in a healthcare setting, humans usually present for care with well advanced disease [111,112]. Combined treatments which reduce viral replication and the host immune response to it are likely to be valuable developments [16].

A wide range of repurposed or novel potential antivirals including polymerase, nucleotide synthesis and protease inhibitors, and fusion-inhibiting peptides [66,91,113,114,115,116,117] have been investigated [91,115]. Corticosteroid use is not recommended for acute respiratory distress syndrome [113]. Comparative studies and randomised controlled trials are mostly lacking [91].

## 8. Future Considerations

Though much is already known, it remains important to clarify the routes of human infection, including the role of the eyes in contracting infection, among primary human cases. The development of rapid molecular POCT tests and alternatives to serology, such as CD8^+^ detection can help us understand MERS-CoV transmission, which can lead to reductions in outbreaks. The scale of mild and subclinical cases among non-hospitalised Arabian Peninsula communities is unknown, as is their role in transmission. Most knowledge of MERS comes from studies of hospital-based populations or linked community contacts. Future prospective long-term cohort studies of mild community respiratory illnesses using molecular methods would be useful. Children have so far been largely absent from the MERS case tally, but they may represent an important population for prospective study. Recent lessons from the Zika and Ebola viruses should also inform new studies seeking possible long-term sequelae and viral persistence and highlight the need to follow-up severe MERS patients.

## 9. Conclusions

In September 2017, MERS-CoV passed its fifth year since discovery. It remains a rare cause of disease in a geographically defined region of the world known for the concurrent presence of infected camel hosts. Much of what we know about MERS relates to severe disease. Travel-related cases continue to challenge the rest of the world’s surveillance and response capabilities, and we need more data to understand unexplained primary transmission. Signs of genetic change in MERS-CoV have been recorded, but it remains unclear whether they change clinical disease. How camels came to carry the virus they live with today remains unknown, but it is academic for the control of MERS. To date, human-to-human transmission has been inefficient, but virus surveillance and characterisation will ensure any change to the status quo is identified. MERS-CoV is not currently a pandemic threat; it is spread with the aid of human habit and error. Nevertheless, a much needed therapeutic toolbox is being developed, and in this process we are learning more about how CoVs cause disease, how they confound our immune responses, where they come from, and how to prevent and treat their respiratory infections [90]. Focused research is needed to minimise the impact of MERS, basing control strategies on evidence gleaned from specifically addressing relevant unknowns.
